# Prevalence and associated factors of high-level diabetes-related distress among patients with type 2 diabetes mellitus in northern Malaysian primary care settings: A cross-sectional study

**DOI:** 10.51866/oa.796

**Published:** 2025-10-05

**Authors:** Peng Yit Tan, Azmilumur Mahfuzah, Kupusamy Gaayatiri, Darshini Latsmanan Rhena, Govindasamy Sangeetha, Mohamed Syarif Mohamed Yassin, Noorhida Baharudin

**Affiliations:** 1 MBBS, FRACGP, Department of Primary Care Medicine, Faculty of Medicine, Universiti Teknologi MARA Sungai Buloh Campus, Sungai Buloh, Selangor, Malaysia.; 2 Cardiovascular Advancement and Research Excellence (CARE) Institute, Universiti Teknologi MARA, Sungai Buloh Campus, Jalan Hospital, Sungai Buloh, Selangor, Malaysia. Email: noorhida8229@uitm.edu.my; 3 MB BCh BAO, Klinik Kesihatan Seberang Jaya, Jalan Perpustakaan, Butterworth, Pulau Pinang, Malaysia.; 4 MBBS, Klinik Kesihatan Kangar, Kangar, Perlis, Malaysia.; 5 MD, Klinik Kesihatan Kulim, Jalan Hospital Lama, Kulim, Kedah, Malaysia.; 6 MBBS, Klinik Kesihatan Kulim, Jalan Hospital Lama, Kulim, Kedah, Malaysia.; 7 MD, Klinik Kesihatan Kulim, Jalan Hospital Lama, Kulim, Kedah, Malaysia.; 8 MBBS, FRACGP, DipPallMed, Department of Primary Care Medicine, Faculty of Medicine, Universiti Teknologi MARA Sungai Buloh Campus, Sungai Buloh, Selangor, Malaysia.

**Keywords:** Type 2 diabetes mellitus, T2DM, Diabetes-related distress, DRD, Malaysia

## Abstract

**Introduction:**

Diabetes-related distress (DRD) affects disease control and quality of life. This study aimed to determine the prevalence of high-level DRD, compare the prevalence according to diabetes complications and identify the associated sociodemographic and clinical factors.

**Methods:**

This cross-sectional study included 198 participants from three primary care clinics in northern Malaysia from 1 May 2023 to 31 July 2023. DRD was measured using the 17-Item Diabetes Distress Scale, with a score of >3 indicating high-level distress. Multiple logistic regression was used to assess the association between the sociodemographic and clinical characteristics and DRD.

**Results:**

The prevalence of high-level DRD was 13.6%. The prevalence was significantly higher among the patients without nephropathy (17.2%) than among those with nephropathy (3.8%) (P=0.014) but did not significantly differ among those with other complications. Those who attended the clinics independently were less likely to be highly distressed than those using public transportation or sent by others (adjusted odds ratio [aOR]=0.31, 95% confidence interval [CI]=0.10–0.99, P=0.048). The participants who were obese (aOR=2.96, 95% CI=1.08-8.08, P=0.034) had higher odds of having high-level DRD than those who were not, while those who had nephropathy (aOR=0.11, 95% CI=0.02-0.58, P=0.010) had lower odds of having high-level DRD than those who did not.

**Conclusion:**

The overall prevalence of high-level DRD is relatively low but significantly higher among individuals without nephropathy. 0besity, lack of transportation and the absence of nephropathy are positively associated with high-level DRD and should therefore be a focus of psychological support and intervention.

## Introduction

Type 2 diabetes mellitus (T2DM) is one of the most prevalent chronic diseases worldwide, affecting approximately 11.1% of the adult population or about one in nine adults globally.^1^ According to the 2023 Malaysian National Health and Morbidity Survey, 15.6% of Malaysian adults aged >18 years had diabetes, with northern states such as Perlis (13.6%) and Pulau Pinang (12.8%) ranking among the top three in diabetes prevalence.^2^ In addition, a large-scale study in Kedah reported that 85% of individuals with diabetes failed to achieve glycaemic targets, primarily due to suboptimal management related to patients’ negative perceptions of treatment, medication refusal and the cost of care.^3^ T2DM poses a detrimental blow and burden on both public health and socioeconomic development, as it causes significant physical and mental impairment to its sufferers.^4,5^ The management of T2DM in primary health facilities has traditionally emphasised glycaemic control and the prevention of anticipated complications. However, less has been done for the psychological aspect of patients who have diabetes.^6^ There are many components of diabetes self-management, such as monitoring one’s blood glucose and maintaining a strict diet, which can be overwhelming for patients. Thus, patients with diabetes may experience psychological distress as a result of having this lifelong condition. The most common psychological disorders experienced by patients with T2DM are diabetes-related distress (DRD) and depressive disorders.^7^

DRD was originally introduced by Polonsky et al. in 1995 as specifically describing the negative emotional experiences that arise from the ongoing challenges and demands of managing life with diabetes.^8^ This construct describes concerns about self-management of diabetes, perception of support, emotional burden and access to quality healthcare among patients with diabetes.^9^ Although related to depression, DRD is conceptually different, as it specifically arises from the daily challenges of managing diabetes, while depression is a broader mental health condition not limited to any specific illness.^10^ The Malaysian and international diabetes guidelines recognise the importance of assessing DRD as an integral part of diabetes management.^11,12^ To facilitate this, healthcare workers can utilise the valid and reliable 17-Item Diabetes Distress Scale (DDS 17) to assess the psychological well-being of patients with diabetes.^13^ Patients who score 3 and above are considered to have a high level of distress necessitating clinical attention.^13^

Several studies have reported that the prevalence of DRD is increasing among patients with diabetes and has a significant impact on glycaemic control.^7,14,15^ With early detection and intervention of respective emotional and psychological distress, patients with diabetes can be treated in a more holistic approach to achieve better glycaemic control and positive longterm outcomes.^14,15^ Jannoo et al. concluded that individuals with higher levels of DRD often show poorer health-related quality of life (HRQoL). The prevalence of DRD was reported to be lower among individuals with better medication adherence and, hence, more promising HRQoL.^16^ Chew et al. found that younger age and Chinese ethnicity were associated with a higher prevalence of DRD. They also suggested that patients’ expectations and demands towards health care services might play a role in DRD.^7^

In terms of the factors associated with DRD, mixed findings have been reported regarding the relationship between DRD and patients’ sociodemographic and clinical characteristics. Stoop et al. found that younger age, ethnic minority status, insulin use, a higher Haemoglobin A1c (HbA1c) level, a higher body mass index (BMI) and the presence of neuropathy were independently associated with DRD.^17^ Chew et al. discovered that the prevalence of DRD was not significantly higher among patients with complications than among those without.^7^ Another study found a significant association between DRD and microvascular complications but not macrovascular complications.^18^ Based on the existing literature, the association between DRD and T2DM complications remains conflicting. Chew et al. hypothesised that this finding could be explained by the lack of heterogeneity of their study population, in which most of their participants did not have any T2DM complications.^7^ Thus, to address this gap, researchers should recruit heterogeneous participants comprising a comparable proportion of those with and without complications to prove the hypothesis that DRD is more prevalent among patients with T2DM complications. Therefore, this study aimed to determine the prevalence of high-level DRD among patients with T2DM, compare the prevalence of high-level DRD between patients with and without diabetes-related complications and identify the sociodemographic and clinical factors associated with high-level DRD among patients with T2DM in three primary health clinics in northern Malaysia.

## Methods

This cross-sectional study was conducted among adult patients with T2DM attending government primary health care clinics in three states (Kedah, Perlis and Penang) in northern Malaysia. The participating clinics were Kulim Health Clinic in Kedah, Kuala Perlis Health Clinic in Perlis and Jalan Angsana Health Clinic in Penang, Malaysia. The sampling frame included patients with T2DM aged 18 years and above who attended these clinics from 1 May 2023 to 31 July 2023.

### Inclusion and exclusion criteria

Patients aged 18 years and above who were diagnosed with T2DM for 1 year or more, had laboratory results within the past year and attended follow-ups at least twice in the past year at either Kulim, Kuala Perlis or Jalan Angsana Health Clinic from 1 May 2023 to 31 July 2023 were included in the study. Conversely, individuals who were pregnant or lactating, those with pre-existing psychiatric disorders that could impair judgement or their ability to respond reliably to the questionnaires, those unable to read or understand English or Malay and non-Malaysian citizens were excluded.

### Variable definition

T2DM was defined as a clinical diagnosis by the clinician and/or the use of any oral hypoglycaemic agents or insulin, as identified in the medical record. Optimal glycaemic control was described as an HbAlc level of ≤7.0%, a level appropriate for most adults, consistent with the recommendation from the local guideline for the management of diabetes.^11^ Complications included at least one of the microvascular (retinopathy, nephropathy or neuropathy) or macrovascular complications (cerebrovascular accident, ischaemic heart disease or peripheral vascular disease, including limb amputation and diabetic foot ulcer) as diagnosed by the clinician. Limb amputation was included as a peripheral vascular disease based on the clinician’s documentation in the medical record, indicating it resulted from ischaemic complications associated with diabetes.

Retinopathy was defined as abnormal fundoscopy findings or a clinician’s diagnosis. Nephropathy was defined as an estimated glomerular filtration rate of less than 60 mL/min/1.73 m^2^, the presence of proteinuria from the urine albumincreatinine ratio or urine full examination and microscopy on at least two occasions or clinician’s diagnosis of nephropathy.^19^ BMI was classified as normal (18.5-22.9 kg/m^2^), overweight (23-27.4 kg/m^2^) or obese (≥27.5 kg/m^2^).^20^ The income categories were based on the Household Income Survey Report 2022 by the Department of Statistics Malaysia.^21^ They included the bottom 40% income group (B40) (less than MYR 5249), the middle 40% income group (M40) (MYR 5250-11,819) and the top 20% income group (T20) (more than MYR 11,819).^21^

### Study tool

The DDS 17 is a self-reported questionnaire used to identify distress among patients with T2DM relative to disease management and control. A Likert scale is used to score each item from 1 *(no problem)* to 6 *(a serious problem).* The total DDS 17 score is obtained, and the mean scores are then calculated. An overall mean score of less than 2 indicates little to no stress; a score of 2-3, moderate distress; and a score of 3 and above, high distress requiring clinical attention.^13,22^ In this study, a score of less than 3 was considered to indicate the absence of high-level DRD. Previous studies have revealed that the DDS 17 is reliable for use in clinical practice and has good sensitivity and specificity.^23^ The Malay version of the DDS 17 was validated in 2015, with satisfactory psychometric properties. It had high internal consistency (Cronbach’s α=0.94), and the 1-month test-retest reliability value (Spearman’s correlation r) was 0.33 (P=0.009).^13^ This study utilised the English and Malay-validated versions of the DDS 17.

### Study conduct: Recruitment and data collection

Medical assistants or staff nurses screened patients who attended the participating clinics for eligibility according to the inclusion and exclusion criteria at the triage counter. Eligible participants were invited, and those interested were given an information sheet regarding the study. They were allowed to clarify any queries with the investigator. The investigator obtained written informed consent before enrolling participants in the study. The questionnaire containing sociodemographic items and the DDS 17 was given to participants. Explicit verbal instruction on how to fill out the form was given. Participants were free to ask for clarification from the researcher when any inquiries arose. Once the questionnaire was completed, participants were requested to return it directly to the researcher. The questionnaire was checked for completeness before participants left the consultation room. The clinical information section was completed by the attending clinical practitioner, who was the investigator of this study. All investigators underwent training before data collection to ensure standardised procedures across all study sites. The convenience sampling method was used until the target sample size was achieved.

### Sample size calculation

As the study both aimed to estimate the prevalence of high-level DRD and compare the prevalence between patients with and without complications, two sample size calculations were performed. The larger calculated sample size (173), based on the comparison between subgroups, was selected to ensure adequate power for both objectives. For the first objective, the single-proportion sample size calculation method was used. According to Chew et al., the prevalence of high-level DRD among patients with T2DM attending government health clinics was 9.9%.^13^ Given an a-value of 0.05 with an absolute precision of 5%, the minimum sample size required was 138.^24^ Considering a nonresponse rate of 20%, the calculated sample size was 166. For the second objective, a sample size calculator was utilised to compare the prevalence of DRD between patients with and without complications.^25^ In their study, Chew et al. reported that the prevalence of high-level DRD among patients with complications was 27.8%, while that among patients without complications was 8.6%.^13^ Based on a study power of 0.8 and a significance level of 0.05 (two-tailed), the calculated sample size was 72 for each group, with a total sample size of 144. Considering a non-response rate of 20%, the calculated sample size required was 173.

### Statistical analysis

There were no missing data. Normally distributed data on the sociodemographic and clinical factors were expressed as means with standard deviations (SDs), while non-normally distributed data were presented as medians with interquartile ranges (IQRs). Frequencies and percentages were used for categorical data. The DDS 17 score was expressed as categorical data. Simple and multiple logistic regression analyses were performed to determine the association between the sociodemographic and clinical factors and high-level DRD. Variables with a P-value of <0.25 were selected for the multiple logistic regression analysis to adjust for potential confounders. P-values of <0.05 were considered statistically significant, with 95% confidence intervals (CIs). The dependent variable was high-level distress (i.e. a mean DDS 17 score of 3 and above). The independent variables were the sociodemographic and clinical characteristics of participants.

## Results

A total of 198 patients were eligible and agreed to participate in this study. The median (IQR) age was 59 (14) years. Among them, 45.5% were men, and 55.5% were women. Most of the participants were Malay (60.6%), followed by Indian (23.2%) and Chinese (15.2%). The majority were Muslim (60.1%), were married (76.8%), had secondary and tertiary education (84.8%) and came from the bottom 40% income group (87.9%) (Table 1).

**Table 1:** Sociodemographic characteristics of the participants (N=198).

	Frequency, n (%)	Median (IQR) or mean (SD)
**Age (years), median (IQR)**		59 (14)
<60	101 (51.0)	
≥60	97 (49.0)	
**Sex**		
Female	108 (55.5)	
Male	90 (45.5)	
**Ethnicity**		
Malay	120 (60.6)	
Indian	46 (23.2)	
Chinese	30 (15.2)	
Others	2 (1.0)	
**Religion**		
Islam	119 (60.1)	
Hindu	44 (22.2)	
Buddhist	29 (14.6)	
Christian	6 (3.0)	
**Marital status**		
Single	8 (4.0)	
Divorced	6 (3.0)	
Widowed	32 (16.2)	
Married	152 (76.8)	
**Educational level**		
No formal education/primary education	30 (15.2)	
Secondary/tertiary education	168 (84.8)	
**Employment status**		
Unemployed/retired	116 (58.6)	
Employed/self-employed	82 (41.4)	
**Income, median (IQR)**		2000 (2325)
Bottom 40% (B40), ≤MYR 5249	174 (87.9)	
Middle 40% (M40), MYR 5250-11,819	23 (11.6)	
Top 20% (T20), >MYR 11,819	1 (0.5)	
**Transportation to the clinic**		
Public transportation/sent by others	48 (24.2)	
Own transportation/walking	150 (75.7)	

* IQR: interquartile range, SD: standard deviation

Table 2 shows the clinical characteristics of the participants. Most of the participants were non-smokers (78.2%) and had hypertension (80.8%) and dyslipidaemia (93.4%). There were 95 participants (48%) with microvascular complications and 31 (15.7%) with macrovascular complications. Approximately 54% were obese. The median (IQR) HbA1c level among the participants was 7.7% (2.7). The mean (SD) systolic blood pressure was 136.36 mmHg (15.7), while the median (IQR) diastolic blood pressure was 81 mmHg (15).

**Table 2:** Clinical characteristics of the participants (N=198).

	Frequency, n (%)	Median (IQR) or mean (SD)
**Smoking status**		
Non-smoker	155 (78.2)	
Ex-smoker	21 (10.6)	
Smoker	22 (11.1)	
**Medication**		
Oral hypoglycaemic agent only	126 (63.6)	
Oral hypoglycaemic agent and insulin	64 (32.3)	
Insulin only	8 (4.0)	
**Comorbidity**		
Hypertension	160 (80.8)	
Dyslipidaemia	185 (93.4)	
IHD	23 (11.6)	
CVA	8 (4.0)	
PVD	1 (0.5)	
**Presence of complications**		
Any diabetes complication	107 (54.0)	
Any macrovascular complication (CVA, IHD or PVD)	31 (15.7)	
Any microvascular complication (retinopathy, neuropathy or nephropathy)	95 (48.0)	
Retinopathy	43 (21.7)	
Nephropathy	53 (26.8)	
Neuropathy	28 (14.1)	
Amputation	3 (1.5)	
Undergoing dialysis	0 (0)	
**Hypoglycaemic episode**		
No	140 (70.7)	
Yes	58 (29.3)	
**Body mass index, median (IQR)**		27.85 (7.3)
Normal (18.5-22.9 kg/m^2^)	23 (11.6)	
Overweight (23.0-27.4 kg/m^2^)	68 (34.3)	
Obese (≥27.5 kg/m^2^)	107 (54.0)	
**HbA1c level (%), median (IQR)**		7.70 (2.7)
**eGFR (mL/min/1.73 m^2^), median (IQR)**		96.0 (29.0)
**Lipid parameters (mmol/L)**		
Total cholesterol level, median (IQR)		4.50 (1.4)
Triglyceride level, median (IQR)		1.40 (0.9)
Low-density lipoprotein level, median (IQR)		2.50 (1.3)
**Blood pressure (mmHg)**		
Systolic blood pressure, mean (SD)		136.36 (15.70)
Diastolic blood pressure, median (IQR)		81.00 (15.0)
**Distress score, mean (SD)**		1.98 (0.87)

IQR: interquartile range, SD: standard deviation, IHD: ischaemic heart disease, CVA: cerebrovascular accident, PVD: peripheral vascular disease, HbA1c: Haemoglobin A1c, eGFR: estimated Glomerular Filtration Rate

**Figure 1:**
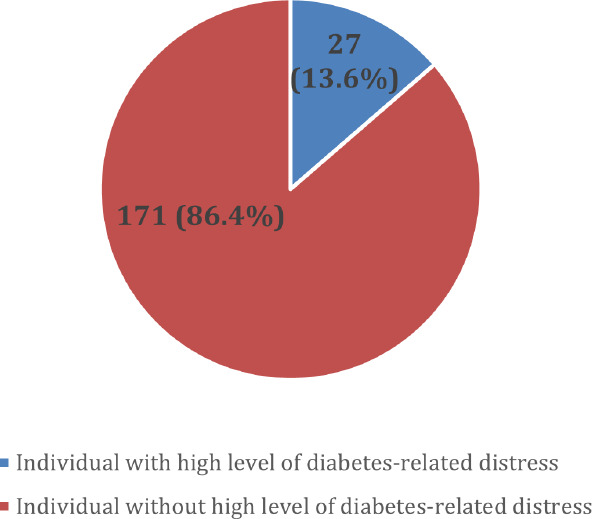
Prevalence of high-level diabetes-related distress.

The mean (SD) distress score was 1.98 (0.87). The prevalence of high-level DRD was 13.6% (Figure 1).

Table 3 presents the prevalence of high-level DRD according to the complications. The prevalence was significantly higher among the patients without nephropathy (17.2%) than among those with nephropathy (3.8%) (P=0.014).

**Table 3:** Prevalence of high-level DRD according to diabetes mellitus complications.

Complication type, n (%)		High-level DRD, n (%)			
		Present	Absent	X^2^ (df)	P-value
Any diabetes complications	Present	12 (11.2)	95 (88.8)	1 .16 (1)	0.282
	Absent	15 (16.5)	76 (83.5)		
Any macrovascular complications (CVA, IHD or PVD)	Present	3 (9.7)	28 (90.3)	0.49 (1)	0.484
	Absent	24 (14.4)	143 (85.6)		
Any microvascular complications (retinopathy, neuropathy or nephropathy)	Present	11 (11.6)	84 (88.4)	0.66 (1)	0.418
	Absent	16 (15.5)	87 (84.5)		
Retinopathy	Present	8 (18.6)	35 (81.4)	1.15 (1)	0.283
	Absent	19 (12.3)	136 (87.7)		
Nephropathy	Present	2 (3.8)	51 (96.2)	5.98 (1)	0.014^*^
	Absent	25 (17.2)	120 (82.8)		
Neuropathy	Present	7 (25.0)	21 (75.0)	3.58 (1)	0.059
	Absent	20 (11.8)	150 (88.2)		

* *Chi-square test, Significant at P<0.05

After adjustment for confounders, the participants who attended the clinics via their own transportation or by walking were found to be less likely to have high-level distress than those who used public transportation or were sent by others (aOR=0.31, P=0.048, 95% CI=0.10-0.99). The participants with nephropathy were less likely to be highly distressed than those without nephropathy (aOR=0.11, P=0.010, 95% CI=0.02-0.58). The obese participants had significantly higher odds of experiencing high-level DRD than the non-obese participants (aOR=2.96, P=0.034, 95% CI=1.08-8.08) (Table 4).

**Table 4:** Factors associated with high-level diabetes-related distress.

	Crude OR (95% CI)	P-value	Adjusted OR (95% CI)	P-value
**Age, year**				
<60	1		1	
≥60	0.47 (0.20-1.11)	0.085	0.51 (0.19-1.42)	0.198
**Sex**				
Female	1		1	
Male	0.56 (0.24-1.31)	0.178	0.78 (0.27-2.19)	0.631
**Ethnicity**				
Non-Malay	1	** **		
Malay	1.12 (0.49-2.60)	0.787	-	-
**Marital status**				
Single/divorced/widowed	1			
Married	0.84 (0.33-2.14)	0.722	-	-
**Educational level**				
No formal education/primary education	1			
Secondary/tertiary education	0.75 (0.26-2.17)	0.601	-	-
**Employment status**				
Unemployed/retired	1			
Employed/self-employed	1.37 (0.61-3.10)	0.446	-	-
**Income**				
B40 (≤MYR 5249)	1		1	
M40 (MYR 5250-11,819)/ T20 (>MYR 11,819)	2.43 (0.87-6.81)	0.091	2.63 (0.76-9.15)	0.127
**Transportation**				
Public transportation/sent by others	1		1	
Own transportation/walking	0.40 (0.17-0.94)	0.035	0.31 (0.10-0.99)	**0.048**
**Smoking status**				
Ex-smoker/non-smoker	1			
Smoker	1.48 (0.46-4.76)	0.512	-	-
**Duration of DM**				
<10 years	1			
≥10 years	0.95 (0.40-2.24)	0.905	-	-
**Polypharmacy (≥5 medications)**				
No	1			
Yes	1.36 (0.54-3.40)	0.517	-	-
**Insulin therapy**				
Without insulin	1		1	
With insulin	1.76 (0.78-4.00)	0.174	1.30 (0.48-3.54)	0.605
**Hypoglycaemic episode**				
No	1			
Yes	0.82 (0.33-2.07)	0.679	-	-
**BMI**				
Non-obese (18.5-27.4 kg/m^2^)	1		1	
Obese (≥27.5 kg/m^2^)	2.76 (1.11-6.86)	0.029	2.96 (1.08-8.08)	0.034
**Hypertension**				
No	1			
Yes	0.81 (0.30-2.16)	0.667	-	-
**Dyslipidaemia**				
No	1			
Yes	0.86 (0.18-4.11)	0.849	-	-
**Presence of any diabetes complications**				
No	1			
Yes	0.64 (0.28-1.45)	0.284	-	-
**Presence of macrovascular complications (CVA, IHD or PVD)**				
No	1			
Yes	0.64 (0.18-2.27)	0.487	-	-
**Presence of microvascular complications (retinopathy, nephropathy or neuropathy)**				
No	1			
Yes	0.71 (0.31-1.62)	0.419	-	-
**Presence of retinopathy**				
No	1			
Yes	1.64 (0.66-4.05)	0.287	-	-
**Presence of nephropathy**				
No	1		1	
Yes	0.19 (0.04-0.82)	0.027	0.11 (0.02-0.58)	**0.010**
**Presence of neuropathy**				
No	1		1	
Yes	2.50 (0.94-6.62)	0.065	3.47 (0.99-12.25)	0.053
**Glycaemic control (HbAlc level of ≤7.0%)**				
Suboptimal	1			
Optimal	0.76 (0.30-1.90)	0.555	-	-
**Control of the low-density lipoprotein level**				
Controlled (≤2.6 mmol/L)	1			
Uncontrolled (>2.6 mmol/L)	0.95 (0.42-2.16)	0.910	-	-
**Control of the triglyceride level**				
Controlled (≤1.7 mmol/L)	1			
Uncontrolled (>1.7 mmol/L)	1.50 (0.64-3.53)	0.353	-	-
**Control of the systolic BP**				
Controlled (<140 mmHg)	1		1	
Uncontrolled (≥140 mmHg)	1.71 (0.76-3.87)	0.196	2.52 (0.96-6.57)	0.059
**Control of the diastolic BP**				
Controlled (<80 mmHg)	1			
Uncontrolled (>80 mmHg)	1.57 (0.68-3.62)	0.293	-	-

1=Reference group. Emboldened: Significant at P<0.05. Variables with P<0.25 were included in the multiple logistic regression: age, sex, income, transportation, BMI, insulin therapy, presence of nephropathy, presence of neuropathy and systolic BP. The model fitted the data well (Hosmer-Lemeshow goodness-of-fit test, P=0.52). Cox and Snell R^2^=14.8%, Nagelkerke R^2^=26.9%. All assumptions (interaction and multicollinearity) were met.

## Discussion

This study determined the prevalence of high-level DRD, compared the prevalence between patients with and without complications and identified the factors associated with high-level DRD. The prevalence of high-level DRD in our study was 13.6%. This rate is slightly higher than that reported in another Malaysian study (9.9%).^13^ In 2022, Liew and Vanoh also found that 7% of their study population in Kelantan, Malaysia, had high levels of DRD.^26^ In contrast, Chew et al. discovered a much higher prevalence of DRD of 49.2% among patients with diabetes attending three public health clinics in the Selangor state of Malaysia in 2016.^7^ The differences in the prevalence among these studies may be due to the different sociodemographic and clinical characteristics of their study populations. Patients with low socioeconomic status and lower levels of education have a poorer understanding of diabetes and its complications. Hence, they are not aware of the complications, leading to low levels of DRD.

We also compared the prevalence of high-level DRD according to the complications. The prevalence of high-level DRD was significantly higher among the individuals without nephropathy (17.2%) than among those with nephropathy (3.8%). Furthermore, the individuals without nephropathy had higher odds of experiencing DRD than those with nephropathy. A plausible explanation is that patients without nephropathy may have greater health awareness, potentially leading to increased anxiety and higher levels of DRD. These individuals might be more health-conscious or concerned about the potential progression of nephropathy, leading to anticipatory anxiety and psychological burden driven by uncertainty about future complications, even in the absence of current ones.^27^ These unmeasured factors, including anticipatory anxiety, depression, perceived disease burden and psychological coping mechanisms, should be considered in future research. Including these variables in the logistic regression analysis could enhance the explanatory power of the current model, as reflected by the Cox and Snell R^2^ of 14.8% and Nagelkerke R^2^ of 26.9%.

In our study, the prevalence of high-level DRD was higher in the retinopathy and neuropathy groups, although the difference was not statistically significant. The patients with retinopathy in this study did not have a complete loss of vision, which may have contributed to them not having significantly high levels of DRD. Furthermore, there was only one participant with peripheral vascular disease. Thus, the level of distress was not significantly higher among those with neuropathy and macrovascular complications.

In terms of the factors associated with high-level DRD, this study found that the participants who were obese had higher odds of having high levels of DRD. This finding is consistent with previous reports.^26,28^ Liew and Vanoh and Aljuaid et al. showed similar results in which BMI was significantly correlated to DRD.^26,28^ A Thai study showed that obesity was linked to poor glycaemic control and subsequently to DRD.^29^ This study also discovered that the participants who needed assistance with their transportation were more likely to have high-level DRD than those who attended the clinics independently. Another local study by Cheng et al. mentioned that a lack of transportation resulted in significant DRD.^30^ Unmet needs regarding transportation to healthcare facilities contributed to DRD, as reported by Thi et al.^31^

Our study also showed no association of macrovascular complications, retinopathy and neuropathy with high-level DRD. This finding is similar to that of another Malaysian study^7^ and could be explained by the lack of knowledge and awareness related to diabetes among the study population.^32^ Having these complications did not affect their psychological health, specifically DRD.

### Strengths, limitations and implications for clinical practice and future research

One strength of this study is the robust sample size calculation for comparing the level of distress between patients with and without complications, addressing a specific gap in the literature by examining whether DRD differs according to complications. The study provides new evidence that DRD levels are significantly higher among patients without nephropathy, a finding that contrasts with some previous assumptions and highlights the complexity of distress in T2DM care. Future research should explore the association of DRD with the severity of complications, such as end-stage nephropathy requiring renal replacement therapy and various severities of retinopathy. This study also recruited participants from three health clinics in northern Malaysia, providing valuable data for this part of the country.

This study has several limitations. The use of convenience sampling may have introduced sampling bias, potentially limiting the representativeness of the sample and, consequently, the generalisability of the findings to the broader population in the states. To address this, we collected data from multiple study sites to reduce location-specific bias and enhance the diversity of the sample. This study used a self-reported questionnaire, which may lead to response bias. The participants could have underreported or overreported their distress due to recall issues or wanting to give socially acceptable answers. This may affect the accuracy of the findings. To reduce this bias, we provided the participants with ample time and privacy to answer the questionnaire. Next, this study utilised regression analysis, which showed only associations without causality. Thus, our results must be interpreted in this context. Additionally, we acknowledge the possibility of a type I error, where some associations may appear statistically significant by chance due to multiple comparisons in the logistic regression analysis. To reduce this risk, we selected the variables based on prior literature demonstrating established significance and scientifically sound hypotheses, rather than relying solely on statistical significance from simple logistic regression. This approach helped minimise false-positive findings and enhance the reliability of the results.

The significant association of obesity, availability of transportation and the absence of nephropathy with high-level DRD has important clinical implications. This finding should alert primary care providers to screen for DRD among at-risk patients. For example, a patient with obesity should be screened for DRD, thus providing them with proactive measures such as monitoring their mental health status and counselling. For patients who have difficulty with transportation to clinics, virtual consultation should be offered, as previous literature has shown that this measure can minimise distress.^33,34^ Patients without nephropathy need to be reassured that they are on the right track with their diabetes management. Psychological support should also be offered to alleviate their distress. Patients identified to be highly distressed should be assessed for depression and anxiety and treated accordingly.

## Conclusion

Although the overall prevalence of high-level DRD is relatively low, it is significantly higher among patients without nephropathy. Obesity, the absence of nephropathy and lack of transportation are significant predictors for DRD. These findings highlight the need to incorporate routine DRD screening into primary care settings, particularly for patients with these risk factors. Practical interventions such as virtual consultations and targeted psychological support may help address this. Given the cross-sectional design of this study, causal relationships could not be established. Therefore, future longitudinal research is recommended to explore the causal relationship between these factors and DRD.

## Data sharing statement

The data are stored at the Department of Primary Care Medicine, Faculty of Medicine, Universiti Teknologi MARA, Malaysia, and will only be shared upon reasonable request to the corresponding author, subject to the data protection regulation.

**How does this paper make a difference in general practice?**

- Patients who are obese, rely on others for transportation and do not have nephropathy have higher odds of having a high level of diabetes-related distress.
- During clinical encounters, these high-risk patients should be screened for distress to facilitate appropriate psychological intervention.
